# Understanding HPC Benchmark Performance on Intel Broadwell and Cascade Lake Processors

**DOI:** 10.1007/978-3-030-50743-5_21

**Published:** 2020-05-22

**Authors:** Christie L. Alappat, Johannes Hofmann, Georg Hager, Holger Fehske, Alan R. Bishop, Gerhard Wellein

**Affiliations:** 8 School of Computing, University of Utah, Salt Lake City, UT USA; 9 Cray, a Hewlett Packard Enterprise Company, Seattle, WA USA; 10 Helmholtz-Zentrum Dresden-Rossendorf, Dresden, Germany; 11 Extreme Computing Research Center, King Abdullah University of Science and Technology, Thuwal, Saudi Arabia; 12 Erlangen Regional Computing Center (RRZE), 91058 Erlangen, Germany; 13 Department of Computer Science, University of Erlangen-Nuremberg, 91058 Erlangen, Germany; 14 Institute of Physics, University of Greifswald, 17489 Greifswald, Germany; 15 Science, Technology and Engineering Directorate, Los Alamos National Laboratory, Los Alamos, USA

**Keywords:** Benchmarking, Microbenchmarking, x86, Intel

## Abstract

Hardware platforms in high performance computing are constantly getting more complex to handle even when considering multicore CPUs alone. Numerous features and configuration options in the hardware and the software environment that are relevant for performance are not even known to most application users or developers. Microbenchmarks, i.e., simple codes that fathom a particular aspect of the hardware, can help to shed light on such issues, but only if they are well understood and if the results can be reconciled with known facts or performance models. The insight gained from microbenchmarks may then be applied to real applications for performance analysis or optimization. In this paper we investigate two modern Intel x86 server CPU architectures in depth: Broadwell EP and Cascade Lake SP. We highlight relevant hardware configuration settings that can have a decisive impact on code performance and show how to properly measure on-chip and off-chip data transfer bandwidths. The new victim L3 cache of Cascade Lake and its advanced replacement policy receive due attention. Finally we use DGEMM, sparse matrix-vector multiplication, and the HPCG benchmark to make a connection to relevant application scenarios.

## Introduction

Over the past few years the field of high performance computing (HPC) has received attention from different vendors, which led to a steep rise in the number of chip architectures. All of these chips have different performance-power-price points, and thus different performance characteristics. This trend is believed to continue in the future with more vendors such as Marvell, Huawei, and Arm entering HPC and related fields with new designs. Benchmarking the architectures to understand their characteristics is pivotal for informed decision making and targeted code optimization. However, with hardware becoming more diverse, proper benchmarking is challenging and error-prone due to wide variety of available but often badly documented tuning knobs and settings.

In this paper we explore two modern Intel server processors, Cascade Lake SP and Broadwell EP, using carefully developed micro-architectural benchmarks, then show how these simple microbenchmark codes become relevant in application scenarios. During the process we demonstrate the different aspects of proper benchmarking like the importance of appropriate tools, the danger of black-box benchmark code, and the influence of different hardware and system settings. We also show how simple performance models can help to draw correct conclusions from the data.

Our microbenchmarking results highlight the changes from the Broadwell to the Cascade Lake architecture and their impact on the performance of HPC applications. Probably the biggest modification in this respect was the introduction of a new L3 cache design.

This paper makes the following relevant contributions:

- We show how proper microarchitectural benchmarking can be used to reveal the cache performance characteristics of modern Intel processors. We compare the performance features of two recent Intel processor generations and resolve inconsistencies in published data.
- We analyze the performance impact of the change in the L3 cache design from Broadwell EP to Skylake/Cascade Lake SP and investigate potential implications for HPC applications (effective L3 size, scalability).
- For DGEMM we show the impact of varying core and Uncore clock speed, problem size, and sub-NUMA clustering on Cascade Lake SP.
- For a series of sparse matrix-vector multiplications we show the consequence of the nonscalable L3 cache and the benefit of the enhanced effective L3 size on Cascade Lake SP.
- To understand the performance characteristics of the HPCG benchmark, we construct and validate the roofline model for all its components and the full solver for the first time. Using the model we identify an MPI desynchronization mechanism in the implementation that causes erratic performance of one solver component.

This paper is organized as follows. After describing the benchmark systems setup in Sect. 2, microarchitectural analysis using microbenchmarks (e.g., load and copy kernels and STREAM) is performed in Sect. 3 to 5. In Sect. 6 we then revisit the findings and see how they affect code from realistic applications. Section 7 concludes the paper.

**Related Work.** There is a vast body of research on benchmarking of HPC systems. The following papers present and analyze microbenchmark and application performance data in order to fathom the capabilities of the hardware.

Molka et al. [17] used their BenchIT microbenchmarking framework to thoroughly analyze latency and bandwidth across the full memory hierarchy of Intel Sandy Bridge and AMD Bulldozer processors, but no application analysis or performance modeling was done. Hofmann et al. [9, 11] presented microbenchmark results for several Intel server CPUs. We extend their methodology towards Cascade Lake SP and also focus on application-near scenarios. Saini et al. [20, 21] compared a range of Intel server processors using diverse microbenchmarks, proxy apps, and application codes. They did not, however, provide a thorough interpretation of the data in terms of the hardware architectures. McIntosh-Smith et al. [15] compared the Marvell ThunderX2 CPU with Intel Broadwell and Skylake using STREAM, proxy apps, and full applications, but without mapping architectural features to microbenchmark experiments. Recently, Hammond et al. [6, 7] performed a benchmark analysis of the Intel Skylake and Marvell ThunderX2 CPUs, presenting results partly in contradiction to known hardware features: Cache bandwidths obtained with standard benchmark tools were too low compared to theoretical limits, the observed memory bandwidth with vectorized vs. scalar STREAM was not interpreted correctly, and matrix-matrix-multiplication performance showed erratic behavior. A deeper investigation of these issues formed the seed for the present paper. Finally, Marjanović et al. [13] attempted a performance model for the HPCG benchmark; we refine and extend their node-level model and validate it with hardware counter data.

## Testbed and Environment

All experiments were carried out on one socket each of Intel’s Broadwell-EP (BDW) and Cascade Lake-SP (CLX) CPUs. These represent previous- and current-generation models in the Intel line of architectures, which encompass more than 85% of the November 2019 top500 list. Table 1 summarizes key specifications of the testbed. Measurements conducted on a Skylake-SP Gold-6148 (SKX) machine are not presented as the results were identical to CLX (successor) in all the cases.

The Broadwell-EP architecture has a three-level inclusive cache hierarchy. The L1 and L2 caches are private to each core and the L3 is shared. BDW supports the AVX2 instruction set, which is capable of 256-bit wide SIMD. The Cascade Lake-SP architecture has a shared non-inclusive victim L3 cache. The particular model in our testbed supports the AVX-512 instruction set and has 512-bit wide SIMD. Both chips support the “Cluster on Die [CoD]” (BDW) or “Sub-NUMA Clustering [SNC]” (CLX) feature, by which the chip can be logically split in two ccNUMA domains.

Unless otherwise specified, hardware prefetchers were enabled. For all microbenchmarks the clock frequency was set to the guaranteed attainable frequency of the processor when all the cores are active, i.e., 1.6 GHz for CLX and 2.0 GHz for BDW. For real application runs, Turbo mode was activated. The Uncore clock speed was always set to the maximum possible frequency of 2.4 GHz on CLX and 2.8 GHz on BDW.

Both systems ran Ubuntu version 18.04.3 (Kernel 4.15.0). The Intel compiler version 19.0 update 2 with the highest optimization flag (-O3) was used throughout. Unless otherwise stated, we added architecture-specific flags -xAVX (-xCORE-AVX512 -qopt-zmm-usage=high) for BDW (CLX). For experiments that use MKL and MPI libraries we used the version that comes bundled with the Intel compiler. The LIKWID tool suite in version 4.3 was used for performance counter measurements and benchmarking (likwid-perfctr and likwid-bench). Note that likwid-bench generates assembly kernels automatically, providing full control over the executed code.

**Table 1.** Key specification of test bed machines.

Microarchitecture	Broadwell-EP (BDW)	Cascade Lake-SP (CLX)
Chip Model	Xeon E5-2697 v4	Xeon Gold 6248
Supported core freqs	1.2–3.6 GHz	1.2–3.9 GHz
Supported Uncore freqs	1.2–2.8 GHz	1.0–2.4 GHz
Cores/Threads	18/36	20/40
Latest SIMD extension	AVX2/FMA	AVX-512
L1 cache capacity	18 $\times$ 32 KiB	20 $\times$ 32 KiB
L2 cache capacity	18 $\times$ 256 KiB	20 $\times$ 1 MiB
L3 cache capacity	45 MiB (18 $\times$ 2.5 MiB)	27.5 MiB (20 $\times$ 1.375 MiB)
Memory Configuration	4 ch. DDR4-2400	6 ch. DDR4-2933
LD/ST throughput	2 LD, 1 ST (AVX)	2 LD, 1 ST (AVX512)
L1 - L2 bandwidth	64 B/cy	64 B/cy
L2 - L3 bandwidth	32 B/cy	16 B/cy + 16 B/cy
Theor. Mem. Bandwidth	76.8 GB/s	140.8 GB/s
Operating system	Ubuntu 18.04.3	Ubuntu 18.04.3
Compiler	Intel 19.0 update 2	Intel 19.0 update 2

**Fig. 1.**
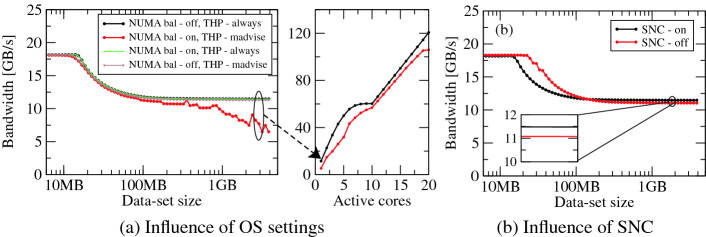
(a) Performance impact of NUMA balancing and transparent huge pages (THP) on a load-only streaming benchmark on CLX. The left figure in (a) shows the single core performance over different data set sizes for various OS settings. The right figure in (a) shows the performance influence of the best and worst setting for different number of cores with a data-set size of 3 GB per core. (b) Performance effect of sub-NUMA clustering (SNC) on single core for the same load-only benchmark. For the experiment in (a) SNC was enabled and in (b) NUMA balancing was disabled and THP set to “always.”

**Influence of Machine and Environment Settings.** The machine and environment settings are a commonly neglected aspect of benchmarking. Since they can have a decisive impact on performance, all available settings must be documented. Figure 1(a) shows the influence of different operating system (OS) settings on a serial load-only benchmark running at 1.6 GHz on CLX for different data-set sizes in L3 and memory. With the default OS setting (NUMA balancing on and transparent huge pages (THP) set to “madvise”), we can see a 2$\times$ hit in performance for big data sets. The influence of these settings can be seen for multi-core runs (see Fig. 1(a) right) where a difference of 12% is observed between the best and default setting on a full socket. This behavior also strongly depends on the OS version. We observed it with Ubuntu 18.04.3 (see Table 1). Consequently, we use the setting that gives highest performance, i.e., NUMA balancing off and THP set to “always,” for all subsequent experiments.

Modern systems have an increasing number of knobs to tune on system startup. Figure 1(b) shows the consequences of the sub-NUMA clustering (SNC) feature on CLX for the load-only benchmark. With SNC active the single core has local access to only one sub-NUMA domain causing the shared L3 size to be halved. For accesses from main memory, disabling SNC slightly reduces the single core performance by 4% as seen in the inset of Fig. 1(b).

## Single-Core Bandwidth Analysis

**Fig. 2.**
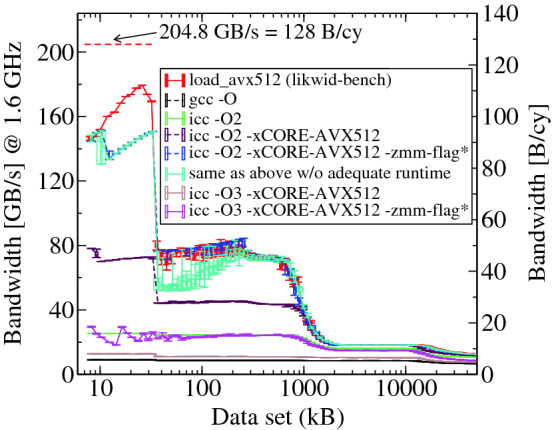
Load-only bandwidth as a function of data set size on CLX. The plot compares the bandwidth obtained from likwid-bench with that of lmbench. likwid-bench is able to achieve 88% of the theoretical L1 bandwidth limit (128 byte/cy). The extreme sensitivity of lmbench benchmark results to compilers and compiler flags is also shown. The “zmm-flag*” refers to the compiler flag -qopt-zmm-usage=high.

Single-core bandwidth analysis is critical to understand the machine characteristics and capability for a wide range of applications, but it requires great care especially when measuring cache bandwidths since any extra cycle will directly change the result. To show this we choose the popular bandwidth measurement tool lmbench [16]. Figure 2 shows the load-only (full-read or frd) bandwidth obtained by lmbench as a function of data set size on CLX at 1.6 GHz. Ten runs per size are presented in a box-and-whisker plot.

Theoretically, one core is capable of two AVX-512 loads per cycle for an L1 bandwidth of 128 byte/cy (204.8 Gbyte/s @ 1.6 GHz). However, with the compiler option -O2 (default setting) it deviates by a huge factor of eight (25.5 Gbyte/s) from the theoretical limit. The characteristic strong performance gap between L1 and L2 is also missing. Therefore, we tested different compiler flags and compilers to see the effect (see Fig. 2) and observed a large span of performance values. Oddly, increasing the level of optimization (-O2 vs -O3) dramatically decreases the performance. The highest bandwidth was attained for -O2 with the architecture-specific flags mentioned in Sect. 2. A deeper investigation reveals that this problem is due to compiler inefficiency and the nature of the benchmark. The frd benchmark performs a sum reduction on an integer array; in the source code, the inner loop is manually unrolled 128 times. With -O2 optimization, the compiler performs exactly 128 ADD operations using eight AVX-512^1^ integer ADD instructions (vpaddd) on eight independent registers. After the loop, a reduction is carried out among these eight registers to accumulate the scalar result. However, with -O3 the compiler performs an additional 16-way unrolling on top of the 128-way manual unrolling and generates sub-optimal code with a long dependency chain and additional instructions (blends, permutations) inside the inner loop, degrading the performance. The run-to-run variability of the highest-performing lmbench variant is also high in the default setting (cyan line). This is due to an inadequate number of warmup runs and repetitions in the default benchmark setting; increasing the default values (to ten warmup runs and 100 repetitions) yields stable measurements (blue line).

We are forced to conclude that the frd benchmark does not allow any profound conclusions about the machine characteristics without a deeper investigation. Thus, lmbench results for frd (e.g., [6, 7, 20, 21]) should be interpreted with due care. However, employing proper tools one can attain bandwidths close to the limits. This is demonstrated by the AVX-512 load-only bandwidth results obtained using likwid-bench [24]. As seen in Fig. 2, with likwid-bench we get 88% of the theoretical limit in L1, the expected drops at the respective cache sizes, and much less run-to-run variations.

**Fig. 3.**
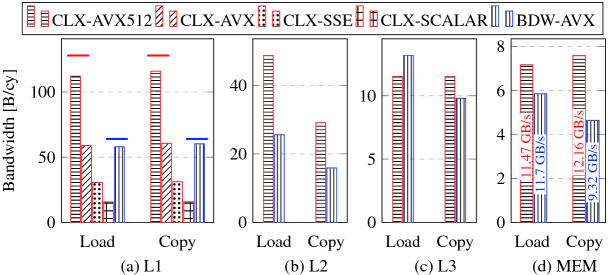
Single-core bandwidth measurements in all memory hierarchy levels for load-only and copy benchmarks (likwid-bench). The bandwidth is shown in byte/cy, which is a frequency-agnostic unit for L1 and L2 cache. For main memory, the bandwidth in Gbyte/s at the base AVX512/AVX clock frequency of 1.6 GHz/2 GHz for CLX/BDW is also indicated. Different SIMD widths are shown for CLX in L1. Horizontal lines denote theoretical upper bandwidth limits.

Figure 3 shows application bandwidths^2^ from different memory hierarchy levels of BDW and CLX (load-only and copy kernels). The core clock frequency was fixed at 1.6 and 2 GHz for CLX and BDW, respectively, with SNC/CoD switched on. The bandwidth is shown in byte/cy, which makes it independent of core clock speed for L1 and L2 caches. Conversion to Gbyte/s is done by multiplying the byte/cy value with the clock frequency in GHz. The effect of single-core L1 bandwidth for scalar and different SIMD width is also shown in Fig. 3(a) for CLX. It can be seen that the bandwidth reduces by 2$\times$ as expected when the SIMD width is halved each time.

## Intel’s New Shared L3 Victim Cache

From BDW to CLX there are no major observable changes to the behavior of L1 and L2 caches, except that the L2 cache size has been significantly extended in CLX. However, starting from Skylake (SKX) the L3 cache has been redesigned. In the following we study the effects of this newly designed non-inclusive victim L3 cache.

### L3 Cache Replacement Policy

A significant change with respect to the L3 cache concerns its replacement policy. Since SNB, which used a pseudo-LRU replacement strategy [1], new Intel microarchitectures have implemented dynamic replacement policies [8] which continuously improved the cache hit rate for streaming workloads from generation to generation. Instead of applying the same pseudo-LRU policy to all workloads, post-SNB processors make use of a small amount of dedicated leader sets, each of which implements a different replacement policy. During execution, the processor constantly monitors which of the leader sets delivers the highest hit rate, and instructs all remaining sets (also called follower sets) to use the best-performing leader set’s replacement strategy [19].

Experimental analysis suggests that the replacement policy selected by the processor for streaming access patterns involves placing new cache lines only in one of the ways of each cache set; the same strategy is used when prefetching data using the prefetchnta instruction (cf. Section 7.6.2.1 in [1]). Consequently, data in the remaining ten ways of the sets will not be preempted and can later be reused.

**Fig. 4.**
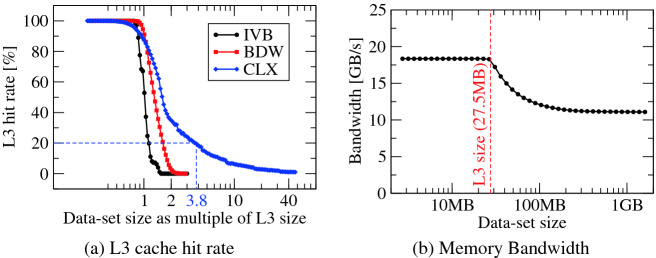
(a) Demonstration of the implications of the change in cache-replacement policy across processor generations using the L3-cache hit rate. (b) Bandwidth for a load-only data-access pattern on CLX (using likwid-bench). In (a), data for the older Intel Ivy Bridge Xeon E5-2690 v2 (IVB) is included for reference.

Figure 4(a) demonstrates the benefit of this replacement policy by comparing it to previous generations’ L3 caches. The figure shows the L3-cache hit rate^3^ for different data-set sizes on different processors for a load-only data access pattern. To put the focus on the impact of the replacement policies on the cache hit rate, hardware prefetchers were disabled during these measurements. Moreover, data-set sizes are normalized to compensate the processors’ different L3-cache capacities. The data indicates that older generations’ L3 caches offer no data reuse for data set sizes of two times the cache capacity, whereas CLX’s L3 delivers hit rates of 20% even for data sets almost four times its capacity. Reuse can by detected even for data sizes more than ten times the L3 cache size on CLX.

The fact that this improvement can also be observed in practice is demonstrated in Fig. 4(b), which shows measured bandwidth for the same load-only data-access pattern on CLX. For this measurement, all hardware prefetchers were enabled. The data indicates that the L3-cache hit-rate improvements directly translate into higher-than-memory bandwidths for data sets well exceeding the L3 cache’s capacity.

### L3 Scalability

Starting from Intel’s Sandy Bridge architecture (created in 2011) the shared L3 cache of all the Intel architectures up to Broadwell is known to scale very well with the number of cores [11]. However, with SKX onwards the L3 cache architecture has changed from the usual ring bus architecture to a mesh architecture. Therefore in this section we test the scalability of this new L3 cache.

In order to test the L3 scalability we use again the likwid-bench tool and run the benchmark with increasing number of cores. The data-set size was carefully chosen to be 2 MB per core to ensure that the size is sufficiently bigger than the L2 cache however small enough such that no significant data traffic is incurred from the main memory.

**Fig. 5.**
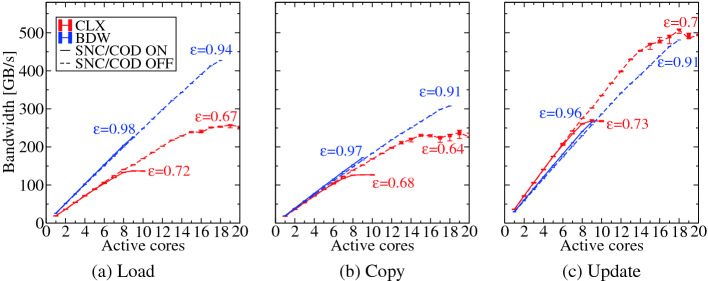
L3 bandwidth of load, copy, and update benchmarks measured on CLX and BDW. The saturation of L3 bandwidth on CLX architecture can be clearly seen. The parallel efficiency of each NUMA domain is further labeled in the plot.

The application bandwidths of the three basic kernels load-only, copy and update are shown in Fig. 5 for CLX and BDW. As the update kernel has equal number of loads and stores it shows the maximum attainable performance on both architectures. Note that also within cache hierarchies write-allocate transfers occur leading to lower copy application bandwidth. The striking difference between CLX and BDW for load-only bandwidth can finally be explained by the bi-directional L2-L3 link on CLX which only has half the load-only bandwidth of BDW (see Table 1).

In terms of scalability we find that the BDW scales almost linearly and attains an efficiency within 90%, proving that the BDW has an almost perfectly scalable L3 cache. However, with CLX this behavior has changed drastically and the L3 cache saturates at higher core counts both with and without SNC enabled, yielding an efficiency of about 70%. Consequently, for applications that employ L3 cache blocking it might be worthwhile to consider L2 blocking instead on SKX and CLX. Applications that use the shared property of L3 cache like some of the temporal blocking schemes [12, 25] might exhibit a similar saturation effect as in Fig. 5.

The effect of SNC/COD mode is also shown in Fig. 5, with dotted lines corresponding to SNC off mode and solid to SNC on mode. For CLX with SNC off mode the bandwidth attained at half of the socket (ten threads) is higher than SNC on mode. This is due to the availability of 2$\times$ more L3 tiles and controllers with SNC off mode.

## Multi-core Scaling with STREAM

The STREAM benchmark [14] measures the achievable memory bandwidth of a processor. Although the code comprises four different loops, their performance is generally similar and usually only the triad (A(:)=B(:)+s*C(:)) is reported. The benchmark output is a bandwidth number in Mbyte/s, assuming 24 byte of data traffic per iteration. The rules state that the working set size should be at least four times the LLC size of the CPU. In the light of the new LLC replacement policies (see Sect. 4.1), this appears too small and we chose a 2 GB working set for our experiments.

Since the target array A causes write misses, the assumption of the benchmark about the code balance is wrong if write-back caches are used and write-allocate transfers cannot be avoided. X86 processors feature *nontemporal store* instructions (also known as *streaming stores*), which bypass the normal cache hierarchy and store into separate write-combine buffers. If a full cache line is to be written, the write-allocate transfer can thus be avoided. Nontemporal stores are only available in SIMD variants on Intel processors, so if the compiler chooses not to use them (or is forced to by a directive or a command line option), write-allocates will occur and the memory bandwidth available to the application is reduced. This is why vectorization *appears* to be linked with better STREAM bandwidth, while it is actually the nontemporal store that cannot be applied for scalar code. Note that a careful investigation of the impact of write-allocate policies is also required on other modern processors such as AMD- or ARM-based systems.^4^

**Fig. 6.**
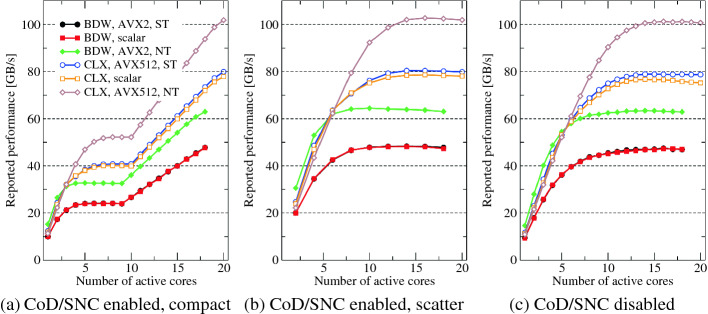
STREAM triad scaling on BDW (closed symbols) and CLX (open symbols) with (a) CoD/SNC enabled and compact pinning of threads to cores, (b) CoD/SNC enabled and scattered pinning of threads to cores, and (c) CoD/SNC disabled. “NT” denotes the use of nontemporal stores (enforced by the -qopt-streaming-stores always), with “ST” the compiler was instructed to avoid them (via -qopt-streaming-stores never), and the “scalar” variant used non-SIMD code (via -no-vec). The working set was 2 GB. Core/Uncore clock speeds were set to 1.6 GHz/2.4 GHz on CLX and 2.0 GHz/2.8 GHz on BDW to make sure that no automatic clock speed reduction can occur. Note that the “scattered” graphs start at two cores.

Figure 6 shows the bandwidth reported by the STREAM triad benchmark on BDW and CLX with (a,b) and without (c) CoD/SNC enabled. There are three data sets in each graph: full vectorization with the widest supported SIMD instruction set and standard stores (ST), scalar code, and full vectorization with nontemporal stores (NT). Note that the scalar and “ST” variants have very similar bandwidth, which is not surprising since they both cause write-allocate transfers for an overall code balance of 32 byte/it. The reported saturated bandwidth of the “NT” variant is higher because the memory interface delivers roughly the same bandwidth but the code balance is only 24 byte/it. This means that the actual bandwidth is the same as the reported bandwidth; with standard stores, it is a factor of 4/3 higher. In case of BDW, the NT store variant thus achieves about the same memory bandwidth as the ST and scalar versions, while on CLX there is a small penalty. Note that earlier Intel processors like Ivy Bridge and Sandy Bridge also cannot attain the same memory bandwidth with NT stores as without. The difference is small enough, however, to still warrant the use of NT stores in performance optimization whenever the store stream(s) require a significant amount of bandwidth.

The peculiar shape of the scaling curve with CoD or SNC enabled and “compact” pinning (filling the physical cores of the socket from left to right, see Fig. 6(a)) is a consequence of the static loop schedule employed by the OpenMP runtime. If only part of the second ccNUMA domain is utilized (i.e., between 10 and 17 cores on BDW and between 11 and 19 cores on CLX), all active cores will have the same workload, but the cores on the first, fully occupied domain have less bandwidth available per core. Due to the implicit barrier at the end of the parallel region, these “slow” cores take longer to do their work than the cores on the other domain. Hence, over the whole runtime of the loop, i.e., including the waiting time at the barrier, each core on the second domain runs at the average performance of a core on the first domain, leading to linear scaling. A “scattered” pinning strategy as shown in Fig. 6(b) has only one saturation curve, of course. Note that the available saturated memory bandwidth is independent of the CoD/SNC setting for both CPUs.

## Implications for Real-World Applications

In the previous sections we discussed microbenchmark analysis of the two Intel architectures. In the following we demonstrate how these results reflect in real applications by investigating important kernels such as DGEMM, sparse matrix-power-vector multiplication, and HPCG. According to settings used in production-level HPC runs, we use Turbo mode and switch off SNC unless specified otherwise. Statistical variations for ten runs are shown whenever the fluctuations are bigger than 5%.

### DGEMM—Double-Precision General Matrix-Matrix Multiplication

If implemented correctly, DGEMM is compute-bound on Intel processors. Each CLX core is capable of executing 32 floating-point operations (flops) per cycle (8 DP numbers per AVX-512 register, 16 flops per fused multiply-add (FMA) instruction, 32 flops using both AVX-512 FMA units). Running DGEMM on all twenty cores, the processor specimen from the testbed managed to sustain a frequency of 2.09 GHz. The upper limit to DGEMM performance is thus 1337.6 Gflop/s.

**Fig. 7.**
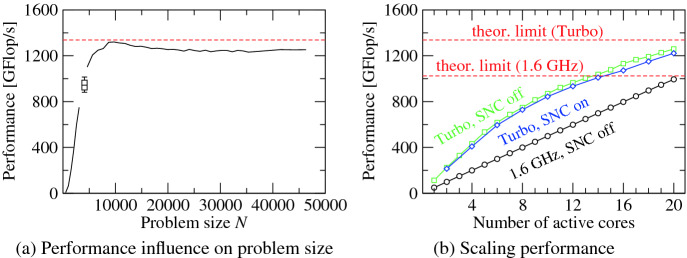
DGEMM performance subject to (a) problem size *N* and (b) number of active cores for $N=40,000$. (Color figure online)

Figure 7(a) compares measured full-chip performance of Intel MKL’s DGEMM implementation on CLX in Turbo mode (black line) to theoretical peak performance (dashed red line). The data indicates that small values of *N* are not suited to produce meaningful results. In addition to resulting in sub-optimal performance, values of *N* below 10,000 lead to significant variance in measurements, as demonstrated for $N=4096$ using a box-plot representation (and reproducing the results from [7]).

Figure 7(b) shows measured DGEMM performance with respect to the number of active cores. When the frequency is fixed (in this case at 1.6 GHz, which is the frequency the processor guarantees to attain when running AVX-512 enabled code on all its cores), DGEMM performance scales all but perfectly with the number of active cores (black line). Consequently, the change of slope in Turbo mode stems solely from a reduction in frequency when increasing the number of active cores. Moreover, the data shows that SNC mode is slightly detrimental to performance (blue vs. green line).

Similar performance behavior can be observed on Haswell-based processors, which have been studied in [10]. However, on Haswell a sensitivity of DGEMM performance to the Uncore frequency could be observed [11]: When running cores in Turbo mode, increasing the Uncore frequency resulted in a decrease of the share of the processor’s TDP available to the cores, which caused them to lower their frequency. On CLX this is no longer the case. Running DGEMM on all cores in Turbo mode results in a clock frequency of 2.09 GHz independent of the Uncore clock. Analysis using hardware events suggests that the Uncore clock is subordinated to the core clock: Using the appropriate MSR (0x620), the Uncore clock can only be increased up to 2.4 GHz. There are, however, no negative consequences of this limitation. Traffic analysis in the memory hierarchy indicates that DGEMM is blocked for the L2 cache, so the Uncore clock (which influences L3 and memory bandwidth) plays no significant role for DGEMM.

### SpMPV – Sparse Matrix-Power-Vector Multiplication

The SpMPV benchmark (see Algorithm 1) computes $y=A^px$, where *A* is a sparse matrix, as a sequence of sparse matrix-vector products. The SpMPV kernel is used in a wide range of numerical algorithms like Chebyshev filter diagonalization for eigenvalue solvers [18], stochastic matrix-function estimators used in big data applications [22], and numerical time propagation [23].

The sparse matrix is stored in the compressed row storage (CRS) format using double precision, and we choose $p=4$ in our experiments. For the basic sparse matrix vector (SpMV) kernel we use the implementation in Intel MKL 19.0.2. The benchmark is repeated multiple times to ensure that it runs for at least one second, so we report the average performance over many runs.

We selected five matrices from the publicly available SuiteSparse Matrix Collection [5]. The choice of matrices was motivated by some of the hardware properties (in particular L3 features) as investigated in previous sections via microbenchmarks. The details of the chosen matrices are listed in Table 2. The matrices were pre-processed with reverse Cuthill-McKee (RCM) to attain better data locality; however, all performance measurements use the pure SpMPV execution time, ignoring the time taken for reordering.
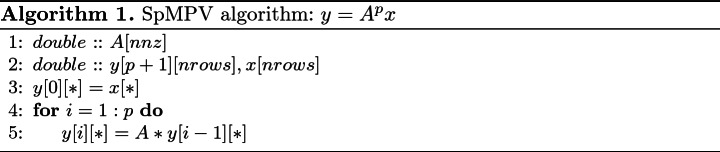

**Table 2.** Details of the benchmark matrices. $N_{\mathrm {r}}$ is the number of matrix rows, $N_{\mathrm {nz}}$ is the number of nonzeros, and $N_{\mathrm {nzr}}=N_\mathrm {nz}/N_\mathrm r$. The last column shows the total memory footprint of the matrix (in CRS storage format).

Index	Matrix name	$N_{\mathrm {r}}$	$N_{\mathrm {nz}}$	$N_{\mathrm {nzr}}$	Size (MB)
1	ct20stif	52,329	2,698,463	52	32
2	boneS01	127,224	6,715,152	53	81
3	ship_003	121,728	8,086,034	66	97
4	pwtk	217,918	11,634,424	53	140
5	dielFilterV3real	1,102,824	89,306,020	81	1072

**Fig. 8.**
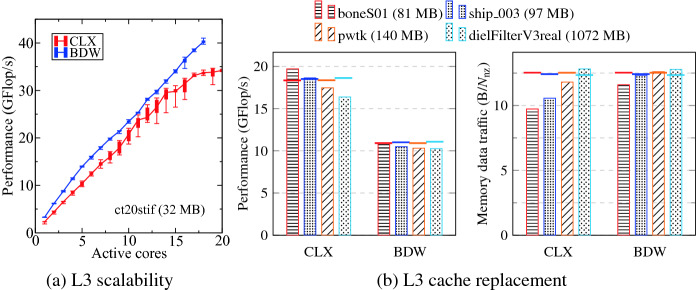
SpMPV benchmark results on CLX and BDW (CoD/SNC off, Turbo mode). (a) Performance for the ct20stif matrix, which fits in the L3 cache. (b) Performance and memory data transfer volume for four different matrices. Dashed lines mark upper limits from a roofline model using the saturated load-only memory bandwidth.

**L3 Scalability.** Figure 8a shows the performance scaling of the ct20stif matrix on CLX and BDW. This matrix is just 32 MB in size and fits easily into the caches of both processors. Note that even though CLX has just 27.5 MiB of L3, it is a non-inclusive victim cache. The applicable cache size using all cores is thus the aggregate L2/L3 cache size, 47.5 MiB. The L3 bandwidth saturation of CLX as shown in Sect. 4.2 is reflected by the performance saturation in the SpMPV benchmark. For this matrix, BDW performs better than CLX since the sparse matrix kernel is predominantly load bound and limited by the bandwidth of the load-only microbenchmark (see Fig. 5a).

Despite this advantage, the in-cache SpMPV scaling on BDW is not linear (parallel efficiency $\varepsilon = 67.5\%$ at all cores), which differs from the microbenchmark results in Fig. 5a. The main reason is the active Turbo mode, causing the clock speed to drop by 25% when using all cores (BDW: 3.6 GHz at single core to 2.7 GHz at full socket; CLX: 3.8 GHz at single core to 2.8 GHz at full socket).

**L3 Cache Replacement Policy.** We have seen in Sect. 4.1 that CLX has a more sophisticated adaptive L3 cache replacement policy, which allows it to extend the caching effect for working sets as big as ten times the cache size. Here we show that SpMPV can profit from this as well. We choose three matrices that are within five times the L3 cache size (index 2, 3, and 4 in Table 2) and a moderately large matrix that is 37 times bigger than the L3 cache (index 5 in Table 2).

Figure 8b shows the full-socket performance and memory transfer volume for the four matrices. Theoretically, with a least-recently used (LRU) policy the benchmark requires a minimum memory data transfer volume of $12+28/N_{\mathrm {nzr}}$ bytes per non-zero entry of the matrix [3]. This lower limit is shown in Fig. 8b (right panel) with dashed lines. We can observe that in some cases the actual memory traffic is lower than the theoretical minimum, because the L3 cache can satisfy some of the cacheline requests. Even though CLX and BDW have almost the same amount of cache, the effect is more prominent on CLX. On BDW it is visible only for the boneS01 matrix, which is 1.7$\times$ bigger than its L3 cache, while on CLX it can be observed even for larger matrices. This is compatible with the microbenchmark results in Sect. 4.1. For some matrices the transfer volume is well below 12 bytes per entry, which indicates that not just the vectors but also some fraction of the matrix stays in cache.

As shown in the left panel of Fig. 8b, the decrease in memory traffic directly leads to higher performance. For two matrices on CLX the performance is higher than the maximum predicted by the roofline model (dashed line) even when using the highest attainable memory bandwidth (load-only). This is in line with data presented in [3].

### HPCG – High Performance Conjugate Gradient

HPCG^5^ (High Performance Conjugate Gradient) is a popular memory-bound proxy application which mimics the behavior of many realistic *sparse* iterative algorithms. However, there has been little work to date on analytic performance modeling of this benchmark. In this section we analyze HPCG using the roofline approach.

The HPCG benchmark implements a preconditioned conjugate gradient (CG) algorithm with a multi-grid (MG) preconditioner. The linear system is derived from a 27-point stencil discretization, but the corresponding sparse matrix is explicitly stored. The benchmark uses the two BLAS-1 kernels DOT and WAXPBY and two kernels (SpMV and MG) involving the sparse matrix. The chip-level performance of HPCG should thus be governed by the memory bandwidth of the processor. Since the benchmark prints the Gflop/s performance of all kernels after a run, this should be straightforward to corroborate. However, the bandwidth varies a lot across different kernels in HPCG (see Table 3): For the WAXPBY kernel (w[i]=a*x[i]+y[i]), which has a code balance of 12 byte/flop^6^, the reported performance is 5.14 Gflop/s on a full socket of BDW. On the other hand, for the DOT kernel with a reported code balance of 8 byte/flop the benchmark reports a performance of 10.16 Gflop/s. According to the roofline model this translates into memory bandwidths of 61.7 Gbyte/s and 81.3 Gbyte/s, respectively. The latter value is substantially higher than any STREAM value presented for BDW in Fig. 6. In the following, we use performance analysis and measurements to explore the cause of this discrepancy, and to check whether the HPCG kernel bandwidths are in line with the microbenchmark analysis.
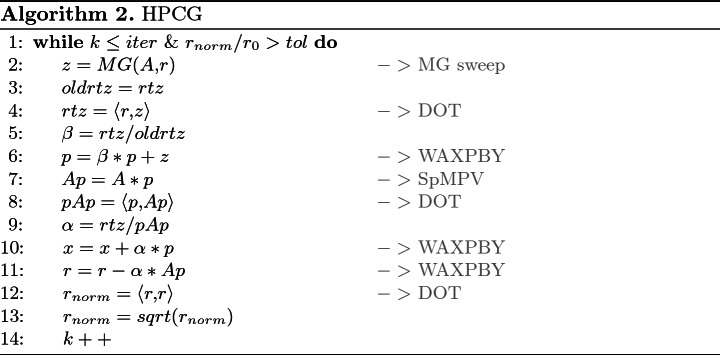


*Setup.* For this analysis we use the recent reference variant of HPCG (version 3.1), which is a straightforward implementation using hybrid MPI+OpenMP parallelization. However, the local symmetric Gauss-Seidel (symGS) smoother used in MG has a distance-1 dependency and is not shared-memory parallel. The main loop of the benchmark is shown in Algorithm 2, where *A* is the sparse matrix stored in CRS format.

As the symGS kernel consumes more than 80% of the entire runtime, the benchmark is run with pure MPI using one process per core. The code implements weak scaling across MPI processes; we choose a local problem size of $160^3$ for a working set of about 1.3 GB per process. The maximum number of CG iteration was set at 25, the highest compiler optimization flag was used (see Table 1), and the contiguous storage of sparse matrix data structures was enabled (-DHPCG_CONTIGUOUS_ARRAYS).

*Performance Analysis of Kernels.* We use the roofline model to model each of the four kernels separately. Due to their strongly memory-bound characteristics, an upper performance limit is given by $P_\mathrm {x}=b_s/C_\mathrm {x}$, where $b_s$ is the full-socket (saturated) memory bandwidth and $C_\mathrm {x}$ is the code balance of the kernel *x*. As we have a mixture of BLAS-1 ($N_{\mathrm {r}}$ iterations) and sparse ($N_{\mathrm {nz}}$ iterations) kernels, $C_x$ is computed in terms of bytes required and work done per row of the matrix.

The reference implementation has three DOT kernels (see Algorithm 2). Two of them need two input vectors (lines 4 and 8 in Algorithm 2) and the other requires just one (norm computation in line 12), resulting in a total average code balance of $C_\mathrm {DOT}$ = $((2\cdot 16 + 8)/3)$ byte/row = 13.3 byte/row. All three WAXPBY kernels need one input vector and one vector to be both loaded and stored, resulting in $C_\mathrm {WAXPBY}$ = 24 byte/row. For sparse kernels, the total data transferred for the inner $N_{\mathrm {nzr}}$ iterations has to be considered. As shown in Sect. 6.2, the optimal code balance for SpMV is $12+28/N_{\mathrm {nzr}}$ bytes per non-zero matrix entry, i.e., $C_\mathrm {SpMV}= (12N_{\mathrm {nzr}}+28)$ byte/row. Note that this is substantially different from the model derived in [13]: We assume that the RHS vector is loaded only once, which makes the model strictly optimistic but is a good approximation for well-structured matrices like the one in HPCG. For the MG preconditioner we consider only the finest grid since the coarse grids do not substantially contribute to the overall runtime. Therefore the MG consists mainly of one symGS pre-smoothing step followed by one SpMV and one symGS post-smoothing step. The symGS comprises a forward sweep (0:nrows) followed by a backward sweep (nrows:0). Both have the same optimal code balance as SpMV, which means that the entire MG operation has a code balance of five times that of SpMV: $C_\mathrm {MG} = 5C_\mathrm {SpMV}$.

The correctness of the predicted code balance can be verified using performance counters. We use the likwid-perfctr tool to count the number of main memory data transfers for each of the kernels.^7^ Table 3 summarizes the predicted and measured code balance values for full-socket execution along with the reported performance and number of flops per row for the four kernels in HPCG. Except for DDOT, the deviation between predicted and measured code balance is less than 10%.

**Fig. 9.**
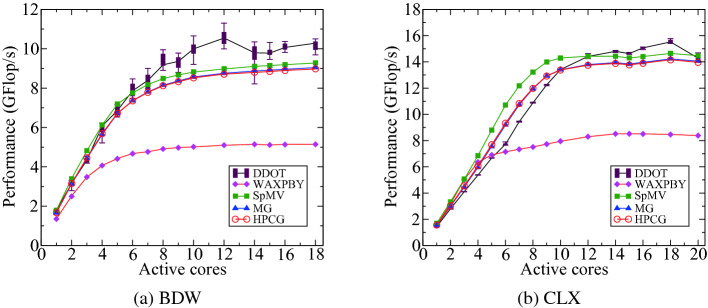
Performance of different kernels in the HPCG benchmark (reference implementation) as a function of active cores.

**Table 3.** Summary of the roofline performance model parameters and measurements for HPCG kernels. Predicted and measured values for code balance and performance are shown in columns three to six. The last two columns compare the predicted and measured performance of the entire solver.

Arch	Kernels	Code balance ($C_\mathrm {x}$)		Performance ($P_\mathrm {x}$)		Flops ($F_\mathrm {x}$)	Calls ($I_\mathrm {x}$)	HPCG perf.	
		Pred.	Measured	Pred.	Measured			Pred.	Measured
		byte/row	byte/row	Gflop/s	Gflop/s	flops/row		Gflop/s	Gflop/s
BDW	DDOT	13.30	11.13	10.23	10.16	2	3	10.27	8.98
	WAXPBY	24.00	24.11	5.67	5.14	2	3		
	SpMV	352.00	385.61	10.43	9.28	54	1		
	MG	1760.00	1952.09	10.43	9.04	270	1		
CLX	DDOT	13.30	12.68	17.29	14.34	2	3	17.37	13.95
	WAXPBY	24.00	24.02	9.58	8.39	2	3		
	SpMV	352.00	382.68	17.64	14.46	54	1		
	MG	1760.00	1944.31	17.64	14.05	270	1		

*MPI Desynchronization.* Surprisingly, DDOT has a measured code balance that is lower than the model, pointing towards caching effects. However, a single input vector for DDOT has a size of 560 MB, which is more than ten times the available cache size. As shown in Sect. 4.1, even CLX is not able to show any significant caching effect with such working sets. Closer investigation revealed *desynchronization* of MPI processes to be the reason for the low code balance: In Algorithm 2 we can see that the DOT kernels can reuse data from previous kernels. For example, the last DOT (line 12) reuses the *r* vector from the preceding WAXPBY. Therefore, if MPI processes desynchronize such that only some of them are already in DOT while the others are still in preceding kernels (like WAXPBY), then the processes in DOT can reuse the data, while the others just need to stream data as there is no reuse. To have a measurable performance impact of the desynchronization phenomenon, a kernel *x* should satisfy the following criteria:

- no global synchronization point between *x* and its preceding kernel(s),
- some of the data used by *x* and its predecessor(s) are the same,
- the common data used by the kernels should have a significant contribution in the code balance ($C_x$) of the kernel.

In Algorithm 2, DOT is the only kernel that satisfies all these conditions and hence it shows the effect of desynchronization.

This desynchronization effect is not predictable and will vary across runs and machines as can be observed in the significant performance fluctuation of DOT in Fig. 9. To verify our assumption we added barriers before the DOT kernels, which caused the measured $C_\mathrm {DOT}$ to go up to 13.3 byte/row, matching the expected value. The desynchronization effect clearly shows the importance of analyzing statistical fluctuations and deviations from performance models. Ignoring them can easily lead to false conclusions about hardware characteristics and code behavior. Desynchronization is a known phenomenon in memory-bound MPI code that can have a decisive influence on performance. See [2] for recent research.

*Combining Kernel Predictions.* Once the performance predictions for individual kernels are in place, we can combine them to get a prediction of the entire HPCG. This is done by using a time-based formulation of the roofline model and linearly combining the predicted kernel runtimes based on their call counts. If $F_\mathrm {x}$ is the number of flops per row and $I_\mathrm {x}$ the number of times the kernel *x* is invoked, the final prediction is

1 $$\begin{aligned} T_\mathrm {HPCG}&= \sum _{x}^{}I_\mathrm {x}T_\mathrm {x} \quad \forall x \in \{\mathrm {DOT, WAXPBY, SpMV, MG}\},\end{aligned}$$

2 $$\begin{aligned} \text {where}\,\,T_\mathrm {x}&= F_\mathrm {x}N_{\mathrm {r}}/P_\mathrm {x}. \end{aligned}$$

Table 3 gives an overview of $F_\mathrm {x}$, $I_\mathrm {x}$, and $C_\mathrm {x}$ for different kernels and compares the predicted and measured performance on a full socket. The prediction is consistently higher than the model because we used the highest attainable bandwidth for the roofline model prediction. For Intel processors this is the load-only bandwidth $b_\mathrm S$ = 115 Gbyte/s (68 Gbyte/s) for CLX (BDW), which is approximately 10% higher than the STREAM values (see Sect. 5). Figure 9 shows the scaling performance of the different kernels in HPCG. The typical saturation pattern of memory-bound code can be observed on both architectures.

## Conclusions and Outlook

Two recent, state-of-the-art generations of Intel architectures have been analyzed: Broadwell EP and Cascade Lake SP. We started with a basic microarchitectural study concentrating on data access. The analysis showed that our benchmarks were able to obtain 85% of the theoretical bandwidth limits. For the first time, the performance effect of Intel’s newly designed shared L3 victim cache was demonstrated. During the process of microbenchmarking we also identified the importance of selecting proper benchmark tools and the impact of various hardware, software, and OS settings, thereby proving the need for detailed documentation. We further demonstrated that the observations made in microbenchmark analysis are well reflected in real-world application scenarios. To this end we investigated the performance characteristics of DGEMM, sparse matrix-vector multiplication, and HPCG. For the first time, a roofline model of HPCG and its components was established and successfully validated for both architectures. Performance modeling was used as a guiding tool throughout this work to get deeper insight and explain anomalies.

Future work will include investigation of benchmarks for random and latency-bound codes along with the development of suitable performance models. The existing and further upcoming wide range of architectures will bring more parameters and benchmarking challenges, which will be very interesting and worthwhile to investigate.

## Footnotes

1 16 integer elements in an AVX512 register.

2 Application bandwidth refers to the bandwidth as seen by the application without the inclusion of hidden data traffic like write-allocate transfers.

3 Based on performance-counter data for the MEM_LOAD_RETIRED_L3_HIT and MISS events.

4 For example, on the Marvell ThunderX2 and many other ARM-based architectures, an automatic detection of streaming patterns can be activated that allows to avoid the write-allocate by claiming cache lines directly at the L2 cache [4].

5 http://www.hpcg-benchmark.org/.

6 The plain WAXPBY kernel has a code balance of 16 byte/flop if a write-allocate transfer must be accounted for; however, in HPCG it is called with w[] and x[] being the same array, so no write-allocate applies.

7 See https://github.com/RRZE-HPC/likwid/wiki/TestAccuracy for validation of the data groups.
